# Blood Antibody Titers and Adverse Reactions after BNT162b2 mRNA Vaccination

**DOI:** 10.3390/vaccines10050640

**Published:** 2022-04-19

**Authors:** Jolanta Kiewisz, Damian Drzyzga, Karolina Rozanska, Emilia Krzynowek, Krzysztof Lukaszuk

**Affiliations:** 1 Department of Human Histology and Embryology, Collegium Medicum, School of Medicine, University of Warmia and Mazury, 10-082 Olsztyn, Poland; jolanta.kiewisz@uwm.edu.pl; 2 INVICTA Research and Development Center, 81-740 Sopot, Poland; karolina.rozanska@invicta.pl (K.R.); emilia.krzynowek@invicta.pl (E.K.); 3 Department of Obstetrics and Gynecology Nursing, Medical University of Gdansk, 80-210 Gdansk, Poland; luka@gumed.edu.pl

**Keywords:** SARS-CoV-2, BNT162b2, mRNA vaccines, antibody response, adverse reactions

## Abstract

This study aimed to measure, considering a prior history of severe acute respiratory syndrome coronavirus 2 (SARS-CoV-2) infection (SCV-negative/positive), antibodies titer using Elecsys Anti-SARS-CoV-2 S immunoassay (Roche Diagnostics, Mannheim, Germany), in a serum of healthcare workers (HCW) who received two doses of BNT162b2 vaccines. The local and systemic adverse reactions occurrence was checked with a self-reported questionnaire. A total of 60 SCV-negative HCW showed lower antibody titers than those presented by SCV-positive subjects (n = 7). The highest antibody level was detected 8 days after the second dose of vaccine administration. At the same time, the titer was higher in the SCV2 -positive than the SCV2-negative group and comparable after the first dose in those who became infected to the level after the second dose of those who did not. The local and systemic effects in the SCV2-negative and SCV2-positive groups appeared independent of the vaccine dose. After the second dose, systemic reactions were reported more often than the local adverse effects. Whether no effect was observed or whether the response was local or systemic, the antibody level in a specific group remains constant. These results can be helpful in the improvement of vaccination programs, controlling the occurrence of adverse and long-term effects of the vaccination.

## 1. Introduction

A severe acute respiratory syndrome coronavirus 2 (SARS-CoV-2), at the time of this study, has infected more than 413 million people and caused the deaths of 5,827,947 million worldwide (https://coronavirus.jhu.edu/map.html accessed on 4 April 2022). The fatality rate of the severe acute respiratory syndrome caused by CoV-2 infection would be around 1%. However, these data are to be taken ”cum grano salis” because the health systems vary from country to country and it is certain that many deaths reported as caused by the SARS-CoV-2 infection are misdiagnosed. Enormous funds have been allocated for research on the pathogenesis, prevention and treatment of the disease caused by SARS-CoV-2. The most powerful method to acquire resistance and prevent the spreading of SARS-CoV-2 is to get vaccinated. Current recommendations are based primarily on pharmaceutical company references that do not intend to individualize the patient. Their goal is to target as many people as possible with vaccination. Thus, it is essential to develop and improve the immunization protocols that will protect the community until the vaccine market is saturated with products manufactured by the pharmaceutical industry.

Generally, the administration of vaccines is associated with the modulation of immunity. Regardless of the type of antigen and formulation used (live, inactivated, mRNA), different antibody titers to protect against the pathogen have been observed, and adverse reactions of varying severity have been reported [1,2,3,4,5]. The first vaccine, which received emergency use authorization by the United States (US) Food and Drug Administration (FDA) in December 2020 to protect against SARS-CoV-2 infection, was a messenger RNA (mRNA) vaccine manufactured by Pfizer/BioNTech (Comirnaty; BNT162b2). Local and systemic adverse events have been reported to this new vaccine [1]. Severe side effects in rare cases were also observed [6].

In most individuals who have not been previously infected with SARS-CoV-2, a positive response is observed 8–14 days after the first dose of the vaccine [7]. Prior SARS-CoV-2 infection modulates the magnitude of the immune response, contributing to increased antibody counts in vaccinated individuals [8,9]. There are already multicenter studies with not more than 280 participants, measuring the humoral response less than and 180 days after vaccine administration, using commercially available kits that detect different types of antibodies recognizing different epitopes of SARS-CoV-2 antigen [5,10,11]. As we do not know how long the long-term humoral response after two doses of the BNT162b2 persists and what are the potential long-term effects of mRNA vaccines, monitoring studies are still needed. Thus, the purpose of this study was to survey the durability of the vaccine-induced immune response in the healthcare workers (HCW) who may or may not have been exposed to the SARS-CoV-2 virus antigen before testing. The tests assessed the blood antibody titers and questionaries were used to identify any adverse events that could then be analyzed in relation to antibody levels. 

## 2. Materials and Methods

### 2.1. Study Population and Vaccination Procedure 

The study was conducted between January 2021 and September 2021 and approved by the Ethics Committee of the Gdansk Regional Medical Board (No. KB-4/21). Informed consent, in written form, was obtained from all participants.

It was an observational prospective cohort study involving 67 HCW who were not dedicated to the care of patients suffer from SARS-CoV-2 infection and were not residents or workers of long-term care facilities. Participants were adults, who were healthy or had a stable chronic medical condition at the moment of vaccination. Exclusion criteria included treatment with immunosuppressive therapy, diagnosis with the immunocompromising condition, or pregnancy. The participants who did not have contact with the SARS-CoV-2 antigen before vaccination were considered a negative group (SCV2-negative; n = 60; mean age: 41.8 years ± 11.2 (mean ± SD); F/M: 52/8). Subjects who reported and/or had been previously infected with SARS-CoV-2, regardless of the time to onset, were considered as SARS-CoV-2 positive (SCV2-positive; n = 7; mean age: 45.6 years ± 9.9 (mean ± SD); F/M: 7/0). We did not exclude any participants from the study group.

Vaccination of study groups was performed with two 30 ug doses of mRNA vaccine (Comirnaty; Pfizer/BioNTech) (BNT162b2). Subjects were tested 21 days after administration of the first dose (at the same time as the second dose) and 8, 14, 30, 90, 120, and 180 days after the second dose. All samples were collected, and measurements were performed immediately. Aliquots of sera were stored frozen at −80 °C.

### 2.2. Antibodies Quantification

The SARS-CoV-2 antibodies were quantified using Elecsys Anti-SARS-CoV-2 S immunoassay (Roche Diagnostics, Mannheim, Germany), which detects IgG and IgM antibodies against the RBD sequence of SARS-CoV-2 spike (S) protein in human serum and plasma. The antibodies number in the tested samples was quantified in units per milliliter (U/mL) and converted to WHO standard (U/mL × 1.029 = BAU_RBD-IgG+IgM_/mL). Antibody response was classified as positive when the titer was ≥0.800 BAU_RBD-IgG+IgM_/mL, according to the manufacturer.

When the detection range of Elecsys Anti-SARS-CoV-2 S immunoassay was exceeded, serum samples were diluted according to the protocol and using the reagents supplied by the manufacturer. 

### 2.3. Documentation

The collected socio-demographic and clinical data included information regarding age, sex, race, body-mass index (BMI), the date of both doses’ vaccinations, any medical conditions, and subscribed medications. Study participants were asked to fulfil the written report about any local, systemic, or adverse reactions following the administration of the BNT162b2, after the first and second dose of the vaccine. Injection site pain, redness, and swelling were classified as local reactions. Fatigue, headache, fever, chills, were categorized as systemic. Adverse reactions like lymphadenopathy, arrhythmia, hemorrhagic stroke, myocardial infarction were also monitored [1].

### 2.4. Statistical Analysis

The statistical analysis was performed using the Python (v. 3.8.5) package SciPy (v. 1.6.1). The difference in antibody levels between the two groups (SCV2-negative and SCV2-positive) was analyzed with the Mann–Whitney U test. The comparison of mean antibody level observed on the day of the second dose and 8 days later in the SCV2-negative and SCV2-positive group were analyzed with paired Student’s *t*-test. Pearson’s χ^2^ test was applied to determine whether there is a statistical difference between nominal variables. We used 95% as well as 99% Bootstrap confidence intervals (CI) to justify the statistical significance between the study groups. A *p*-value < 0.05 was considered statistically significant and the exact values were reported in the text unless *p* < 0.001.

## 3. Results

### 3.1. Baseline Metrics of Study Participants

Demographics and vaccine responsiveness of participants who met the study inclusion and exclusion criteria are shown in Table 1. 

### 3.2. Dynamics of the Immune Response in SCV2-Negative and SCV2-Positive Patients after the Second Dose of the BNT162b2 Vaccine

Out of 60 double-dose vaccinated subjects who were SCV2-negative, there were no individuals whose antibody level did not rise. 

**Figure 1:**
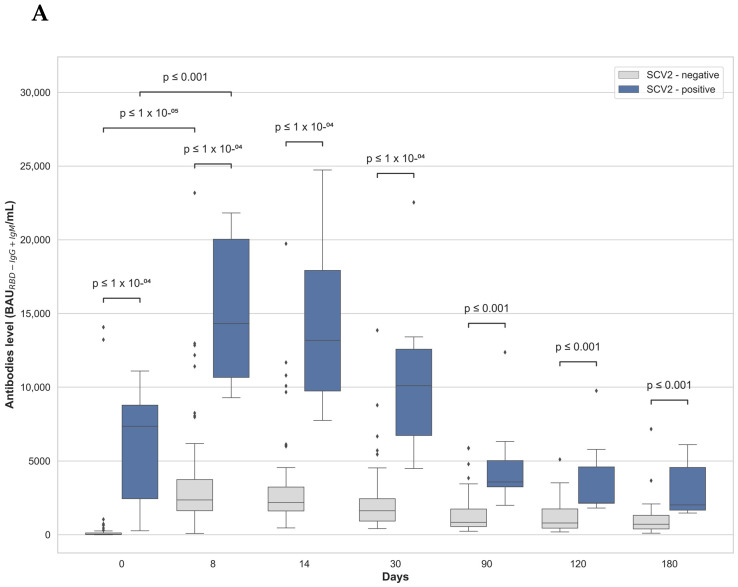

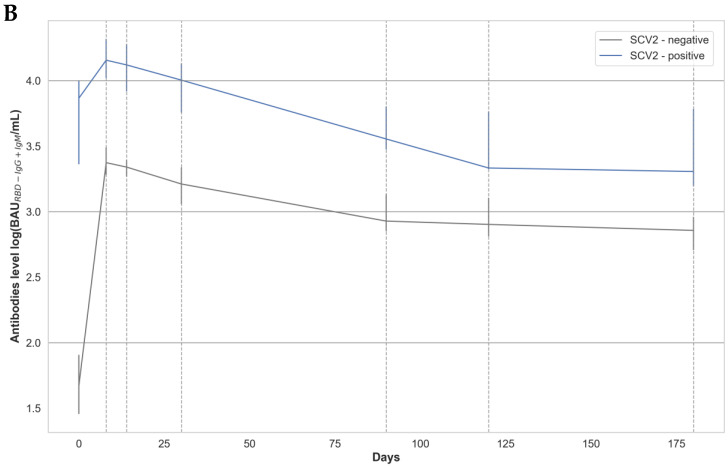
The logarithm value of median antibody levels (**A**) and the trend of changes of antibody levels (**B**) in the SCV2-negative and SCV2-positive groups, on different days, after the second dose of vaccine. For each box, the horizontal line inside the box shows the median. The ends of the box represent the 1st and 3rd quartiles, and the distance between them is called the interquartile range (IQR). The lower whisker end corresponds to a Q1–1.5 IQR value that is not an outlier observation; the upper whisker end, Q3 + 1.5 IQR, is expressed as the highest non-outlier observation.

A significantly higher antibody level was detected in the SCV2-positive compared to the SCV2-negative group, at all time points, after two vaccine doses (Figure 1 A,B). The antibody level in the SCV2-negative and the SCV2-positive group on the day of the second dose of vaccine was 1145.0 (451.9–2172.0) BAU_RBD-IgG+IgM_/mL and 7139.0 (2926.0–10830.0) BAU_RBD-IgG+IgM_ /mL, respectively. Moreover, there was a statistically significant difference between the mean antibody level observed on the day of the second dose and 8 days later in the SCV2-negative (*p* < 1 × 10^−5^) and SCV2-positive group (*p* < 0.001). The rate of change of the mean antibody value between the day of the second vaccine dose administration and 8 days after was higher in the SCV-negative (at day 0: 559.17 BAU_RBD-IgG+IgM_/mL and day 8: 3601.91 BAU_RBD-IgG+IgM_/mL; 6.44 fold) than the SCV-positive group (day 0: 5718.00 BAU_RBD-IgG+IgM_ /mL and day 8: 14834.14 BAU_RBD-IgG+IgM_/mL; 2.59 fold). 

The number, the percentage of participants, and the mean antibody level of SCV2-negative and SCV2-positive groups after the second dose of vaccine, classified into groups: (i) <100 BAU_RBD-IgG+IgM_ /mL; (ii) 101–1000 BAU_RBD-IgG+IgM_ /mL; (iii) 1000–2500 BAU_RBD-IgG+IgM_ /mL; (iv) >2500 BAU_RBD-IgG+IgM_ /mL, at different timelines, are presented in Table 2.

### 3.3. Incidence of Local, Systemic, and Severe Adverse Effects after Administration of a First and Second Dose of BNT162b2 Vaccine in the SCV2-Negative and SCV2-Positive Patients

The appearance of specific local and systemic effects, after the first and second vaccine dose in the SCV2-negative and the SCV2-positive groups, is presented in Table 3.

**Figure 2:**
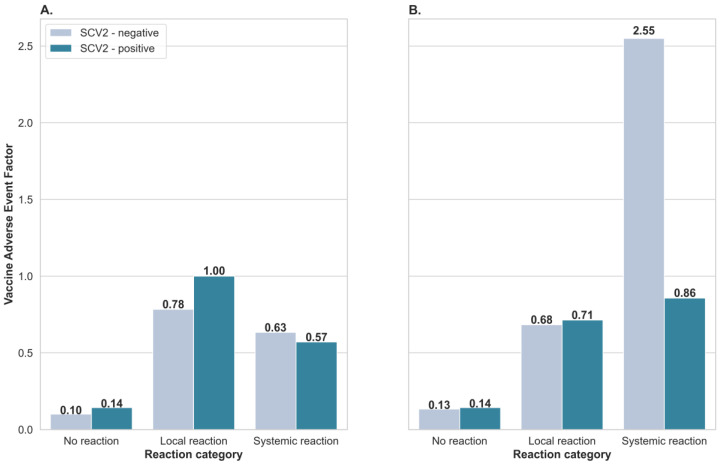
Comparison of Vaccine Adverse Event Factor after first (**A**) and second (**B**) vaccine doses, between participants presenting no reaction, local reaction, or systemic reaction in SCV2-negative and SCV2-positive groups, after administration of the BNT162b2 vaccine.

To estimate the appearance of incidence of the adverse event following immunization per 1 person, classified as local or systemic reactions in the SCV2-negative and SCV2-positive groups, we have calculated the Vaccine Adverse Event Factor (VAEF; Figure 2). The VAEF factor was calculated after dividing the number corresponding to a specific local or systemic adverse reaction by the number of participants in the group presenting a specific response. After the first vaccine dose, the local adverse reactions were more frequent than systemic adverse reactions. Furthermore, they occurred more often in the SCV2-positive (VAEF = 1) than in the SCV2-negative group (VAEF = 0.78; Figure 2A). After the second vaccine dose, the systemic effect was more common than the local adverse effect. Moreover, it appears more often in the SCV2-negative (VAEF = 2.55) than the SCV2-positive group (VAEF = 0.86; Figure 2B).

The Chi-square statistic test was applied to measure the independence of variables with respect to vaccine dose and observed antibody reaction between SCV2 groups. Obtained *p*-values for the first dose (*p* = 0.85) and second dose (*p* = 0.14) of the vaccination subset indicate the variables’ dependence has not been shown, which confirms that adverse effects occur regardless of whether the first or second dose of the vaccine is given.

**Figure 3:**
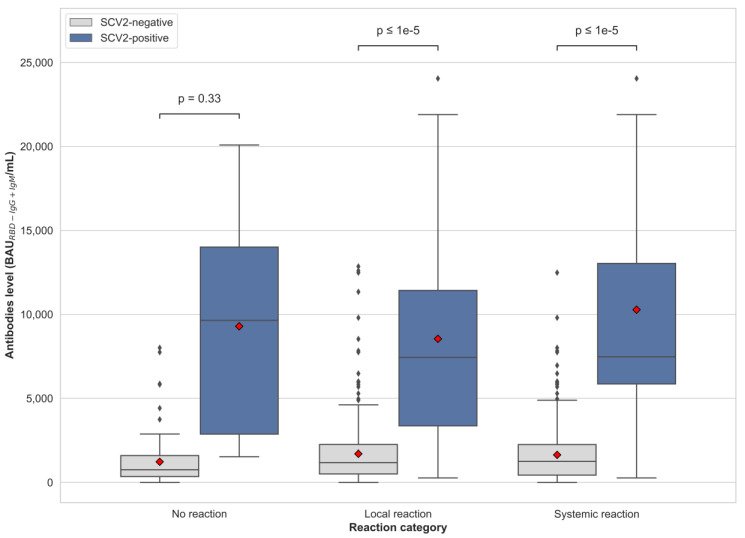
Antibody titer of anti-SARS-CoV2 antibody of patients who did not suffer any adverse reactions and those who presented local and systemic adverse reactions in SCV2-negative and SCV2-positive group, after the second vaccine dose. The red rhombus indicates the mean antibody level. For each box, the horizontal line inside the box shows the median. The ends of the box represent the 1st and 3rd quartiles, and the distance between them is called the interquartile range (IQR). The lower whisker end corresponds to a Q1− 1.5 IQR value that is not an outlier observation; the upper whisker end Q3 + 1.5 IQR is expressed as the highest non-outlier observation.

We observed that the antibody titer is lower in the SCV2-negative in comparison to the SCV2-positive group after the second vaccine dose, no matter if the patients presented no reactions (*p* < 0.33) or local (*p* < 0.0001) or systemic adverse reactions (*p* < 0.0001; Figure 3). 

## 4. Discussion 

The present study demonstrates higher antibody levels in the SCV2-positive group compared to the SCV-negative group on the day of the second dose and at all scheduled time points after the second dose. It confirms the findings of other studies where the antibody level was higher in subjects who had had a previous SARS-CoV-2 infection [10,11,12,13,14]. It can be explained by the fact that after 1–3 days following vaccine application, pyrogenic and inflammatory cytokines (e.g., interleukin-1, interleukin-6, and tumor necrosis factor) from APC (f. e. macrophages) and dendritic cells can pass the antigenic information to relevant immune cells [4]. Cells, like antigen-specific antibody-producing B-cells, start to produce antibodies [4]. Intense production of antibodies by B lymphocytes can also take place after contact with the antigen. Thus, the observed higher antibody level in the SCV2-positive group is due to the overlapping effect of antibodies produced after infection and vaccination. In this way, vaccine and natural immunity combine to form what is known as “hybrid immunity”, providing broader cross-protection against different pathogen variants [15]. 

The presented findings show a dynamic increase in antibody level in both SCV2-negative and SCV2-positive groups on the eighth day post-vaccination, which is in agreement with other studies, where the rise of level of antibodies was detected between days 8–12 [7]. Opposite to the presented data, Mahallawi and coworkers [8] observed no statistically significant increase in antibody titers 2–3 weeks after vaccination, with the first compared to the second vaccine dose in patients who were previously SARS-CoV-2 positive. Moreover, according to [8], the antibody titer in the blood of infection-naive participants who received two vaccine doses was similar to the level observed in individuals who recovered from SARS-CoV-2 infection and were after a single dose of the vaccine. It is suspected that the observed differences were due to the use of a different sensitivity laboratory assay, different antibody types and usage by the Mahallawi group [8], collected approximately 2–3 weeks after vaccination, whereas we compared samples collected from SCV2-positive participants who are no less than 21 days after the first dose and SCV2-negative participants who were 8 days after the second doses.

In our study, blood antibody levels begin to decline between 30 and 90 days and light decline persists until 180 days after the second dose of vaccine in both the SCV2-negative and SCV2-positive groups. The use of ECLIA is maintained for IgG and IgM detection. Thus, the observed decline of the estimated antibody values is not surprising, as it is due to IgG levels dropping over time. Ponticelli observed that the level of antibodies after vaccination decreased after two months [11]; while [5] detected a reduction of the antibody amount between days 21 and 114 regardless of the participant status. The data presented here are consistent with those presented by [5], who observed a similar decrease in total antibody levels in seronegative and seropositive SARS-CoV-2 participants. Moreover, we do confirm the observation that the BNT162b2 vaccine effectiveness against SARS-CoV-2 infection persists till 180 days [16]. It is also similar to the status of subjects injected with the second dose of mRNA Moderna vaccine whose antibody level persists for 6 months [17].

When the BNT162b2 vaccine was monitored during clinical trials, about 80% of participants reported at least one adverse reaction [1,18]. It was also observed that after administrating the second dose, the frequency of adverse reaction appearance was at 70% and was higher in comparison to the 41% observed after the first dose [3]. It is evident also from our studies that adverse reactions occur regardless of which dose of vaccine was administrated. Moreover, we observed that patients who have already suffered from SARS-CoV-2 infection (SCV2-positive group) hardly complained of systemic vaccine reactions after the first vaccination dose but experienced local and systemic reactions after the second dose. In other words, local reactions were more common than systemic adverse reactions after administration of the first dose. The situation is completely different for patients in the SCV2-negative group who have experienced both local and systemic reactions after the first dose. It was already shown that about 96% of patients with a history of severe SARS-CoV-2 disease reported adverse effects on receiving the vaccine [3]. The frequency of adverse reactions was also higher in the SARS-CoV-2 positive group in comparison to those groups where patients have no history of SARS-CoV-2 infection [3]. More severe systemic reactions after the second vaccine dose in SCV-2 serum negative HCWs have been already reported [19]. 

We observed in our study that the occurrence of adverse effects is not correlated with antibody levels, regardless of whether the individual is SCV2 positive or SCV2 negative. In the BNT162b2-vaccinated HCW group, the difference in the reaction was age-dependent [2,19] and weight dependent [19], and no correlation between antibody level and appearance of vaccine-induced symptoms has been reported [19,20]. Additionally, we have observed the most common reactions observed by the others like pain (local reaction [2,3]), fatigue, muscle ache, headache, chills and malaise (systemic reactions [2,3]). However, as far as we know, vaccine-associated adverse reactions are still less commonly reported in the subjects vaccinated with BNT162b2 than in those administrated with a ChAdOx1 nCoV-19 [2] and mRNA-1273 vaccine [3].

As with the majority of studies, the design of the current study is subject to limitations. The first is the limited number of participants. However, those who attempted were not nominated but chosen from a randomized group, which excluded subjective selection. The second limitation concerns the lack of exclusion of the asymptomatic patients at the moment of injection due to no previous PCR test. It disturbs the establishment of the basic level of BNT162b2 vaccine-induced immunogenicity, and analyzed and explained the cases in which a low antibody level is observed. To monitor the humoral response, we used specific and sensitive antibody assays. However, we did not investigate the neutralization antibody titers, due to the lack of a biosafety level 3 (BSL3) laboratory. Moreover, the cellular immune response was not measured. The immune system reacts to physiological changes occurring depending on age and gender which influence the number and type of antibodies in the blood. However, we did not check specific classes which were affected during vaccinal stimulation.

## 5. Conclusions

Vaccination of the population is now the only solution leading to a return to the life that was normal before the pandemic. The vaccines that have been developed and approved for use show the promised efficacy in many independent studies. However, widely published adverse event data have shown a high rate of incidence of adverse events following administration of the anty-SARS-CoV-2 vaccine compared to other approved viral vaccines (for example, against HPV) [21]. Moreover, due to reported rare cases of blood clots after administration of mRNA-containing vaccines (specifically, those based on adenoviral vectors [22] and allergic reactions reported after mass vaccination (https://apps.who.int/iris/bitstream/handle/10665/338096/WHO-2019-nCoV-vaccines-SAGE_evaluation-BNT162b2–2020.1-eng.pdf?sequence=1&isAllowed=y accessed on 4 April 2022), personalized clinical precautions should be taken. The evolving pandemic situation and emerging new mutations of the viral genome continue to pose a threat and require vigilance. 

## Figures and Tables

**Table 1:** Demographic characteristics and response to the vaccine in the studied population.

Stage	Population Characteristic	SCV2-Negative Group	SCV2-Positive Group
Patient characteristic	Sex (F/M)	52/8	7/0
	Age (years; mean ± SD)	41.8 ± 11.2	45.6 ± 9.9
	BMI ^§^	24.5 ± 4.3	27.4 ± 6.3
	BMI ≥30	5	3
	Comorbidities (no./%)	26/43.3	5/71.4
	Supplementation of medicaments (no./%)	25/41.7	3/42.9
1st dose of the vaccine	All observed local reactions (no.)	47	7
	Participants presenting at least one local reaction (no./%)	37/61.7	6/85.7
	All observed systemic reactions (no)	38	4
	Participants presenting at least one systemic reaction (no./%)	18/30	2/28.6
	Adverse reaction (no./%)	0	0
	No reaction (no./%)	6/10.0	1/14.3
2nd dose of the vaccine	Median antibody level BAU ^§§^_RBD-IgG+IgM_/mL	1145.0 (451.9–2172.0)	7139.0 (2926.0–10,830.0)
	All observed local reactions (no.)	41	5
	Participants presenting at least one local reaction (no./%)	34/56.7	5/71.4
	All observed systemic reactions (no)	153	6
	Participants presenting at least one systemic reaction (no./%)	37/61.7	3/42.9
	Adverse reaction (no./%)	0	0
	No reaction (no./%)	8/13.3	1/14.3

^§^ BMI (Body Mass Index) kg/m^2; **§§**^ BAU (Binding Antibody Units) WHO standard for Elecsys Anti-SARS-CoV-2 S immunoassay (U/mL × 1.029 = BAU_RBD-IgG+IgM_/mL).

**Table 2:** The number, the percentage of participants, and the mean antibody (Ab) level at SCV2-negative and SCV2-positive group after the second vaccine dose.

	N	Ab Concentration (BAU_RBD-IgG+IgM_ /mL) ^§^	8 Day		14 Day		30 Day		90 Day		120 Day		180 Day	
			N/%	Mean Ab Value ± SD	N/%	Mean Ab Value ± SD	N/%	Mean Ab Value ± SD	N/%	Mean Ab Value ± SD	N/%	Mean Ab Value ± SD	N/%	Mean Ab Value ± SD
**SCV2-negative**	**60**	**<100**	1.0/1.7	85.5 ± 0.0	0.0/0.0	0.0 ± 0.0	0.0/0.0	0.0 ± 0.0	0.0/0.0	0.0 ± 0.0	0.0/0.0	0.0 ± 0.0	0.0/0.0	0.0 ± 0.0
		**101–1000**	5.0/8.3	735.6 ± 158.9	4.0/6.7	582.3 ± 86.3	19.0/31.7	714.3 ± 177.4	34.0/56.7	568.8 ± 200.6	35.0/58.3	522.8 ± 221.0	40.0/66.7	481.9 ± 246.5
		**1000–2500**	28.0/46.7	1806.1 ± 397.8	36.0/60.0	1786.6 ± 456.9	28.0/46.7	1759.5 ± 481.6	17.0/28.3	1638.8 ± 402.3	20.0/33.3	1689.9 ± 397.6	18.0/30.0	1534.1 ± 318.4
		**>2500**	26.0/43.3	6222.3 ± 4620.5	20.0/33.3	5995.0 ± 4149.1	13.0/21.7	5034.2 ± 3092.9	9.0/15.0	3877.6 ± 1177.4	5.0/8.3	3324.2 ± 974.2	2.0/3.3	5261.0 ± 2394.3
**SCV2-positive**	**7**	**<100**	0.0/0.0	0.0 ± 0.0	0.0/0.0	0.0 ± 0.0	0.0/0.0	0.0 ± 0.0	0.0/0.0	0.0 ± 0.0	0.0/0.0	0.0 ± 0.0	0.0/0.0	0.0 ± 0.0
		**101–1000**	0.0/0.0	0.0 ± 0.0	0.0/0.0	0.0 ± 0.0	0.0/0.0	0.0 ± 0.0	0.0/0.0	0.0 ± 0.0	0.0/0.0	0.0 ± 0.0	0.0/0.0	0.0 ± 0.0
		**1000–2500**	0.0/0.0	0.0 ± 0.0	0.0/0.0	0.0 ± 0.0	0.0/0.0	0.0 ± 0.0	1.0/14.3	1943.0 ± 0.0	4.0/57.1	2001.2 ± 159.3	4.0/57.1	1663.8 ± 235.4
		**>2500**	7.0/100.0	14,834.1 ± 5149.5	7.0/100.0	14,021.0 ± 5969.0	7.0/100.0	10,513.4 ± 5895.1	6.0/85.7	5268.5 ± 3497.8	3.0/42.9	6143.0 ± 3108.6	3.0/42.9	4937.3 ± 1660.0

**^§^** BAU (Binding Antibody Units) WHO standard for Elecsys Anti-SARS-CoV-2 S immunoassay (U/mL × 1.029 = BAU_RBD-IgG+IgM_/mL).

**Table 3:** The number of self-reported local and systemic effects, in SCV2-negative and SCV2-positive groups, after the first and second vaccine dose.

Reaction	Characteristic	SCV-Negative		SCV-Positive	
		1st Dose (N/% Group)	2nd Dose (N/% Group)	1st Dose (N/% Group)	2nd Dose (N/% Group)
**Local**	Redness and swelling at the injection site	23/38.3	17/28.3	3/43.9	1/14.3
	Pain at the injection site	24/40.0	24/40.0	3/43.9	4/57.1
**Systemic**	Fatigue/ Tiredness	5/8.3	22/36.7	2/28.6	2/28.6
	Headache	8/13.3	21/35.0	0/0	0/0
	Fever	3/5.0	1/1.7	0/0	1/14.3
	Chills	5/8.3	21/35.0	0/0	1/14.3
	Diarrhea	0/0.0	4/6.7	0/0	0/0
	Nausea	1/1.7	2/3.3	0/0	0/0
	Muscles pains	2/3.3	24/40.0	0/0	2/28.6

## Data Availability

The data presented in this study are available on request from the corresponding author.
